# Physiological and Molecular Processes Associated with Long Duration of ABA Treatment

**DOI:** 10.3389/fpls.2018.00176

**Published:** 2018-02-21

**Authors:** Mei Wang, Juhun Lee, Bongsoo Choi, Youngmin Park, Hee-Jung Sim, Hyeran Kim, Inhwan Hwang

**Affiliations:** ^1^Key Laboratory of Plant Cell Engineering and Germplasm Innovation, Ministry of Education, School of Life Science, Shandong University, Jinan, China; ^2^Division of Integrative Biosciences and Biotechnology, Pohang University of Science and Technology, Pohang, South Korea; ^3^Center for Genome Engineering, Institute for Basic Science, Daejeon, South Korea; ^4^Environmental Toxicology Research Center, Gyeongnam Department of Environmental Toxicology and Chemistry, Korea Institute of Toxicology, Jinju, South Korea; ^5^Department of Biological Sciences, Kangwon National University, Chuncheon, South Korea; ^6^Department of Life Sciences, Pohang University of Science and Technology, Pohang, South Korea

**Keywords:** ABA response, chlorophyll, chloroplast, long term ABA effect, photosynthesis, short term ABA effect, transitional response

## Abstract

Plants need to respond to various environmental stresses such as abiotic stress for proper development and growth. The responses to abiotic stress can be biochemically demanding, resulting in a trade-off that negatively affects plant growth and development. Thus, plant stress responses must be fine-tuned depending on the stress severity and duration. Abscisic acid, a phytohormone, plays a key role in responses to abiotic stress. Here, we investigated time-dependent physiological and molecular responses to long-term ABA treatment in Arabidopsis as an approach to gain insight into the plant responses to long-term abiotic stress. Upon ABA treatment, the amount of cellular ABA increased to higher levels, reaching to a peak at 24 h after treatment (HAT), and then gradually decreased with time whereas ABA-GE was maintained at lower levels until 24 HAT and then abruptly increased to higher levels at 48 HAT followed by a gradual decline at later time points. Many genes involved in dehydration stress responses, ABA metabolism, chloroplast biogenesis, and chlorophyll degradation were strongly expressed at early time points with a peak at 24 or 48 HAT followed by gradual decreases in induction fold or even suppression at later time points. At the physiological level, long-term ABA treatment caused leaf yellowing, reduced chlorophyll levels, and inhibited chloroplast division in addition to the growth suppression whereas short-term ABA treatment did not affect chlorophyll levels. Our results indicate that the duration of ABA treatment is a crucial factor in determining the mode of ABA-mediated signaling and plant responses: active mobilization of cellular resources at early time points and suppressive responses at later time points.

## Introduction

Plants have evolved complex signaling pathways to respond and adapt to changes in environmental conditions. Water is one of the most crucial natural resources for plant growth and reproduction (Morison et al., 2008). Thus, plants must have a large number of mechanisms to respond to water-related environmental conditions such as dehydration and osmotic stress (Boudsocq and Laurie‘re, 2005; Hanin et al., 2011). Understanding dehydration or osmotic stress responses becomes particularly challenging because stress severity and duration continuously change (Ambrosone et al., 2017). In addition, the amount of water required by plants also varies depending on plant growth and development. For example, the cellular water content is substantially lower during the late stages of seed production in seed plants than during normal vegetative growth (Baud et al., 2002). This suggests that plants must have highly complex mechanisms to control water usage according to the demands of developmental programs and also to coordinate the development and growth with environmental conditions in water availability.

A great deal of studies have been performed to investigate how plants respond to dehydration or osmotic stress, and these studies have yielded significant advances in basic research and agricultural applications (Vinocur and Altman, 2005). Now it is clear that plants contain a large number of mechanisms to cope with continuous changes in water availability (Ingram and Bartels, 1996; Osakabe et al., 2013). In these mechanisms, the signaling mediated by abscisic acid (ABA), a phytohormone, constitutes a core component (Zhu, 2002; Lim et al., 2015). Cellular ABA levels fluctuate depending on intrinsic developmental programs and abiotic stress conditions. High ABA levels lead to the induction of genes with crucial roles in embryogenesis and responses to abiotic stress such as dehydration. ABA must be maintained at lower levels in germinating seeds for efficient germination.

Abscisic acid levels in plant cells can be increased by *de novo* biosynthesis or via hydrolysis of the inactive glucose-conjugated form (ABA-GE) to ABA by β-glucosidases (Lee et al., 2006; Xu et al., 2012). The *de novo* ABA biosynthetic pathway has been clarified using mutants with specific defects at each step along the pathway (Milborrow, 2001; Finkelstein, 2013). The *de novo* ABA biosynthesis pathway involves two different cellular compartments and many intermediates. The last two steps of the pathway occur in the cytosol, whereas all other steps occur in the plastid. Previous work identified two pathways catalyzed by the AtBG1 and AtBG2 β-glucosidases, which produce ABA via hydrolysis of glucose from ABA-GE (Lee et al., 2006; Xu et al., 2012). These reactions take place in the endoplasmic reticulum (ER) or vacuole. Thus, ABA biosynthetic pathways involve multiple organelles (Finkelstein, 2013). By contrast, ABA levels can be reduced by catabolic pathways (Kushiro et al., 2004; Dong et al., 2014; Liu et al., 2015). One major catabolic pathway involves ABA hydroxylation at the 7′ or 8′ position by the cytosolic cytochrome P450-type hydroxylases CYP707A1, CYP707A2, CYP707A3, or CYP707A4. The hydroxylated ABA is further processed through spontaneous conversion to phaseic acid (Kushiro et al., 2004; Finkelstein, 2013). Eventually, this pathway leads to ABA degradation (Endo et al., 2011). In another catabolic pathway, the UDP ABA-glucosyltransferases conjugate glucose to ABA to generate the inactive ABA-GE form (Priest et al., 2006; Dong et al., 2014; Liu et al., 2015). In addition, cellular ABA levels also are regulated by transporters at the plasma membrane (Kuromori et al., 2010; Kang et al., 2010, 2015). Several transporters have been identified that function in ABA export and import out of and into cells, respectively, depending on environmental and intrinsic cellular conditions (Park et al., 2016). Dehydration or osmotic stress conditions activate biosynthetic genes to increase cellular ABA levels. Intriguingly, dehydration or osmotic stress conditions also activate catabolic pathways, although the reason for this apparent paradox is not clearly understood (Xiong and Zhu, 2003).

Extensive studies have been carried out to understand the mechanisms by which ABA-mediated signaling contributes to plant responses to abiotic stresses such as dehydration and osmotic stresses at the molecular and physiological levels (Tuteja, 2007). ABA initiates the signaling by binding to ABA receptors (Ma et al., 2009; Park et al., 2009). Plants contain multiple types of ABA receptors. Of these ABA receptors, the cytosolic receptors, Pyrabactin Resistant (PYR)/PYR-Like (PYL)/Regulatory Component of ABA Receptor (RCAR) proteins, have been studied in detail for the action mechanism. Binding of ABA to the cytosolic receptors leads to inhibition of PP2Cs, the negative regulator of ABA signaling, via a direct interaction between ABA-bound PYR/PYL/RCARs and PP2Cs. Inhibition of PP2Cs results in the activation of Sucrose Non-fermentation Kinase Subfamily 2 (SnRK2s) protein kinases (Hubbard et al., 2010). The activation of SnRK2s induces a large number of cellular responses including stomatal closure to prevent water loss and the expression of many genes whose products are important for stress responses and tolerance such as enzymes for osmoprotectant synthesis (Fujita et al., 2009). Eventually these responses contribute to the enhanced resistance to abiotic stress.

The duration of abiotic stress such as dehydration stress can vary from hours to months or longer under the natural field conditions. The plant responses to abiotic stress likely vary depending on the duration of stress. Consistent with this notion, a recent study showed that distinct gene networks drive differential response to abrupt or gradual long-term water deficit in potato (Ambrosone et al., 2017). In addition, the degree of stress varies continuously in the field. In this study, to gain insight into the mechanism of how plants differentially respond to abiotic stress depending on the duration of the stress, we used the long-term ABA treatment as a means to induce abiotic stress responses in Arabidopsis. ABA was applied to Arabidopsis exogenously, and then the plant responses to ABA were recorded at different time points during 11 days of ABA treatment. Here, we present evidence that plants display a transitional response to exogenously applied ABA over time. Chlorophyll levels were not affected during the first 24 h after ABA treatment, but gradually declined thereafter. At the molecular level, the expression of genes involved in dehydration stress responses, chloroplast biogenesis, ABA biosynthesis, and chlorophyll degradation was strongly induced during the first 24 ∼ 48 h after ABA treatment, but gradually declined or became suppressed thereafter.

## Materials and Methods

### Plant Growth, ABA and NaCl Treatment Conditions

Arabidopsis (*Arabidopsis thaliana*) seeds (ecotype Col-0) were sown on 0.8% w/v agar plates containing half-strength Murashige and Skoog (½ MS) and 2 mM MES (pH 5.7) (not containing sucrose), and kept at 4°C in the dark for 3 days. The plates were transferred to a growth chamber with 70–80 μmol m^-2^ s^-1^ light, a 16 h light/8 h dark cycle, and 22 ± 1°C/16 ± 4°C day/night cycles. For growth measurements, seedlings grown on ½ MS agar plates were transferred onto ½ MS agar plates supplemented with various concentrations of ABA or DMSO as a control. Biomass, root growth, and chlorophyll contents were measured at different time points after transplantation. To determine the expression patterns of chloroplast development-related genes and ABA metabolism-related genes, 12-day-old seedlings grown on ½ MS agar plates were treated with DMSO or 5 μM ABA for the indicated times. To test the expression level of chloroplast- and osmotic stress-related genes under the NaCl-treated condition, 8-day-old seedlings grown on ½ MS (not containing sucrose) agar plates were transferred to ½ MS (not containing sucrose) agar plates or ½ MS agar plates supplemented with 160 mM NaCl for indicated periods of time. To examine the expression of chloroplast development-related genes in response to different concentrations of ABA, 8-day-old seedlings were transferred onto B5 medium (containing 2% sucrose) supplemented with DMSO, or 10 or 100 μM ABA, and treated for 1, 3, 5, and 11 days, and the expression levels of *PORA*, *HEMA1*, *GLK1*, and *GUN4* were determined at different time points.

### Quantitative Real-Time PCR (qRT-PCR) Analysis

Total RNA was extracted from 30 seedlings using an RNA queous phenol-free total RNA isolation kit (Ambion) and treated with TURBO DNase (Invitrogen). 2 μg of RNA were reverse-transcribed into cDNA using a high-capacity cDNA reverse transcription kit (Applied Biosystems). The cDNAs were used as template for quantitative real-time PCR (qRT-PCR) with Power SYBR Green PCR Master Mix (Applied Biosystems). *ACT2* was used as an internal control. Gene-specific primer sequences are listed in Supplementary Table 1. All reactions were run in triplicate. *P*-values were calculated using Student’s *t*-test. Single and double asterisks indicate significant differences as determined by Student’s *t*-test at *P* < 0.05 and *P* < 0.01, respectively.

### ABA and ABA-GE Quantification by Liquid Chromatography (LC)/Mass Spectrometry (MS)

A modification of the method of Park et al. (2016) was used to determine ABA and ABA-GE contents. Briefly, 8-day-old seedlings grown on ½ MS media (not containing sucrose) were transferred to 5 μM ABA-containing media and treated for 12, 24, 48, 72, or 120 h, or transferred onto DMSO, or transferred onto 10, 50, or 100 μM ABA-containing medium and grown for an additional 5 or 11 days. A total of 0.1-0.2 g of frozen fresh sample was ground in liquid nitrogen with a small steel ball in a 2 mL vial. Following the addition of 1.0 mL of ethyl acetate, homogenates were mixed in a Geno/Grinder homogenizer. After centrifugation at 15,200 ×*g* for 10 min at 4°C, the supernatant was transferred to a 2 mL Eppendorf tube. After the second extraction by adding 0.5 mL of ethyl acetate without internal standards, the combined extracts were vacuum-dried in a concentrator at 30°C. The dried extracts were dissolved in 100 μL of 70% methanol, vortexed for 20 min, and then centrifuged at 15,200 ×*g* for 10 min at 4°C. The supernatant was transferred to 1.5 mL LC vials, and then injected into the liquid chromatography (LC)/mass spectrometry (MS) system.

### UPLC/MS Conditions for Quantification of Phytohormones

Ultra-performance liquid chromatography (UPLC) analysis was performed using an ACQUITYVRUPLC system (Waters Corp., Milford, MA, United States) coupled to a Q-TOF instrument (XEVO G2XS; Waters Corp.). The chromatographic separation was carried out on an ACQUITY UPLC BEH C18 column (100 mm × 2.1 mm, i.d., 1.7 μm) connected to an ACQUITY UPLC BEH C18 VanGuard pre-column (5 mm × 2.1 mm, i.d., 1.7 μm). The mobile phases consisted of solvent A (0.1% formic acid) and solvent B (acetonitrile). The gradient elution mode was programmed as follow: 5–60% B for 0.0–7.5 min and 60–95% B for 7.5–10.0 min. The column was then washed with 95% B for 3 min and equilibrated with 5% B for 2 min. All samples were kept at 10°C during the analysis. The flow rate and injection volume were 0.4 mL/min and 2 μL, respectively. MS analysis was conducted in the negative ion mode with electrospray ionization (ESI). The MS conditions were optimized as follows; capillary voltage, 3 kV; cone voltage, 40 V; source temperature, 130°C; desolvation temperature, 400°C; cone gas flow, 50 L/h; desolvation gas flow, 900 L/h.

### Measurement of Chlorophyll Contents

Chlorophylls were extracted from seedling leaf tissues using 50 volumes of 95% (v/v) ethanol at 4°C overnight in the dark. Chlorophyll a/b contents were measured using optical density (OD) at 664 and 648 nm (Vernon, 1960). Chlorophyll a contents were calculated as 5.24 × OD_664_/20. Chlorophyll b contents were calculated as 22.24 × OD_648_/20. Total chlorophyll contents are the sum of chlorophyll a and b contents.

### Transmission Electron Microscopy (TEM)

Leaf tissues of plants treated with ABA or DMSO were harvested and fixed using 2% paraformaldehyde and 2% glutaraldehyde in 0.05 M sodium cacodylate buffer (pH 7.2) at 4°C for 2-4 h, followed by washing with 0.05 M sodium cacodylate buffer (pH 7.2) at 4°C three times. Samples were post-fixed using 1% osmium tetroxide in 0.05 M sodium cacodylate buffer (pH 7.2) at 4°C for 2 h, followed by washing with distilled water three times at room temperature. Sample blocks were stained using 0.5% uranyl acetate at 4°C for 30 min or overnight, dehydrated serially using 30, 50, 70, 80, 90, and 100% ethanol at room temperature for 10 min, and finally substituted twice with 100% propylene oxide at room temperature for 15 min. Finally, samples were embedded with a mixture of propylene oxide:Spurr’s resin at a 2:1 ratio for 1 h, 1:1 ratio for 1 h, 1:2 ratio for 2 h, 0:1 ratio for 4 h or overnight, and 0:1 ratio for 2 h. The samples were polymerized at 70°C for 24 h, sectioned using an ultramicrotome (MT-X, RMC, Tucson, AZ, United States), stained with 2% uranyl acetate for 7 min and Reynolds’ lead citrate for 2 min, and observed by transmission electron microscopy (TEM; JEM-1011, JEOL, Tokyo, Japan).

### Chloroplast Counting

Chloroplast numbers per cell were determined as described by Pyke and Leech (1991). Ten-day-old seedlings grown on ½ MS plates (without sucrose) were transferred to ½ MS plates supplemented with DMSO or 10 μM ABA, and grown for an additional 15 days. The first or second true leaves were fixed with 3.5% glutaraldehyde for 1 h in the dark. The leaf tissue was washed once, and then incubated with 0.1 M Na_2_-EDTA (pH 9.0) for 3.5 h at 60°C to soften the tissue. The samples were macerated on a microscope slide and analyzed using light microscopy.

### Photosynthetic Efficiency Measurement

Maximal photosystem II quantum yield was used as a proxy indicator of photosynthetic efficiency (Maxwell and Johnson, 2000). Chlorophyll fluorescence was measured using an IMAGING-PAM M-series Chlorophyll Fluorometer (Heinz Walz GmbH, Germany) at 30 min after dark adaptation.

### Statistical Analysis

The data are reported in figures as means with standard deviation (SD)or standard error (SE) from three independent experiments. Statistical analysis was performed using SAS 9.2 (SAS Institute). Means were compared using Student’s *t*-test. Asterisks in the figures denote significant differences as follows: ^∗^*P* < 0.05, ^∗∗^*P* < 0.01.

## Result

### Long-term ABA Treatment Attenuates Plant Growth and Development

**FIGURE 1:**
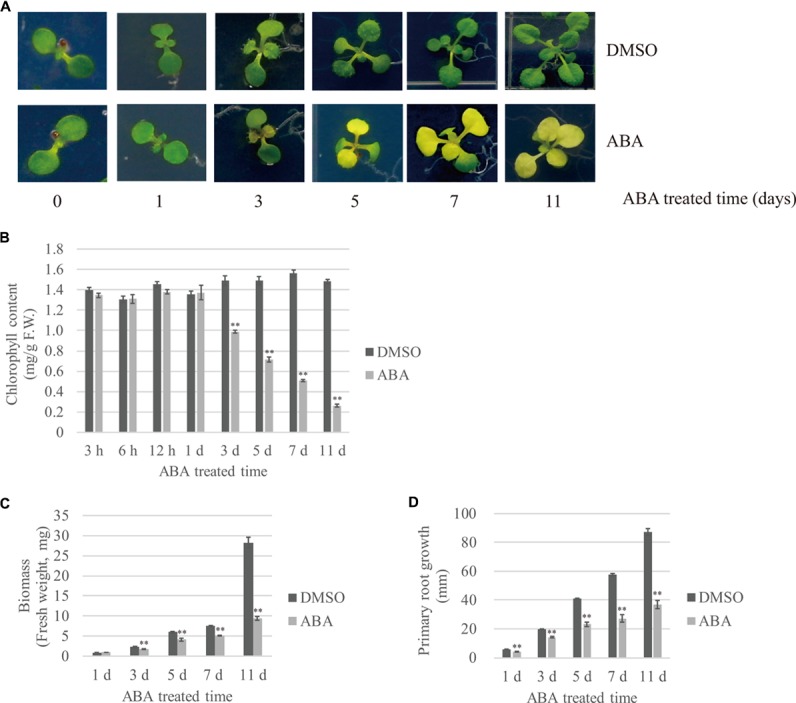
Effects of exogenously applied ABA on temporal patterns of plant growth and development. Plants grown on ½ MS plates for 7–8 days were transferred onto ½ MS plates supplemented with DMSO or 10 μM ABA, and further grown for the indicated periods of time. The effect of ABA on plant growth was monitored using the following growth parameters: **(A)** Overall plant morphology, **(B)** Chlorophyll contents. Shoot tissues (20 ∼ 30 mg) were used in each measurement. Data were obtained using three biological replicates. Error bar indicates standard error (SE), **(C)** Biomass of whole plants and **(D)** Primary root length. 20 seedlings were analyzed. Error bar indicates SE.

To gain insight into plant responses to prolonged abiotic stress, we subjected plants to long-term ABA treatment and monitored their responses at several time points. ABA is a phytohormone that plays a key role in abiotic stress-resistant responses (Hubbard et al., 2010; Kim et al., 2010). Moreover, exogenously applied ABA alone can induce many aspects of molecular and physiological responses to abiotic stresses (Tuteja, 2007). Previous studies have tended to treat plants with ABA for short periods of time (e.g., 30 min to several hours) and then examined the expression patterns of ABA-inducible genes. To examine the effects of long-term ABA treatment during vegetative growth, 7- to 8-day-old plants were transferred onto ½ MS plates supplemented with 10 μM ABA or DMSO as a control, and plant responses to ABA were monitored at different time points during 11 days after transfer (DAT). The most prominent visible changes were yellowing and reduced growth of true leaves. Previous studies show that ABA induces leaf senescence, which can lead to leaf yellowing (Zhao et al., 2016). The Arabidopsis seedlings had two true leaves at the time of transfer; these leaves did grow larger after ABA treatment, but started to turn yellow at 3 DAT. Subsequently emerged true leaves also showed leaf yellowing (**Figure 1A**). To quantify the degree of leaf yellowing, we measured the chlorophyll content at various time points. The chlorophyll content was unchanged by ABA treatment during the 1st day after transfer; however, the chlorophyll content significantly declined after ABA treatment starting at 3 DAT, and was reduced to 17% of the control at 11 DAT (**Figure 1B**). Cotyledons displayed different responses to ABA depending on the plant age; the cotyledons of 7- to 8-day-old seedlings treated with ABA stayed green until 11 DAT, whereas 15-day-old plants displayed senesced cotyledons at 11 DAT (Supplementary Figure 1). Next, we assessed the effect of ABA on plant growth by quantifying plant fresh weight (FW) and root length over time. In the presence of ABA, the above-ground biomass and root length were reduced to 36 and 42% (**Figures 1C,D**), respectively, of that of DMSO controls, indicating that ABA treatment severely attenuates plant growth.

### ABA Treatment Reduces the Leaf Chlorophyll Content

**FIGURE 2:**
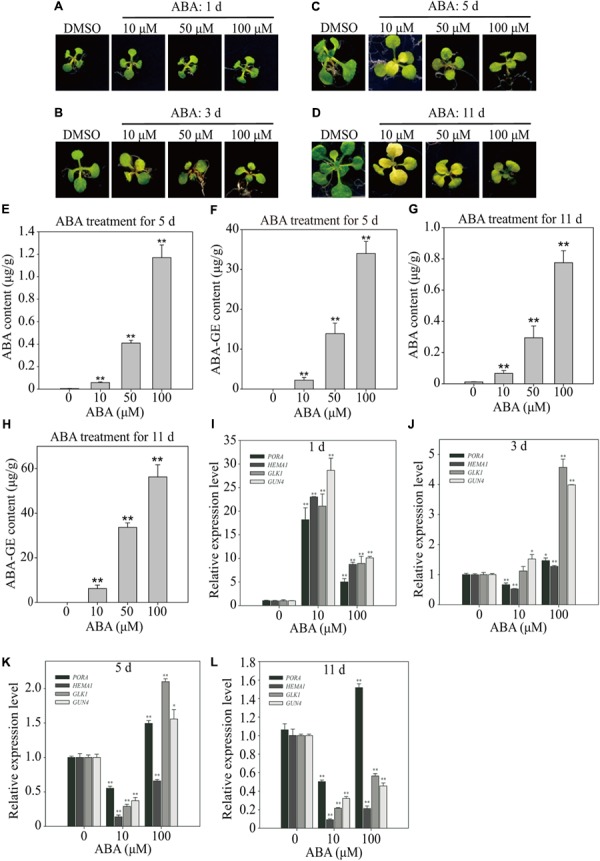
Effect of exogenously applied ABA on endogenous ABA levels and expression of chloroplast development-related genes. **(A–D)** Phenotype of 8-day-old seedlings grown on ½ MS medium supplemented with DMSO or different concentrations of ABA for 1 day **(A)**, 3 days **(B)**, 5 days **(C)**, and 11 days **(D)**. **(E–H)** Endogenous ABA and ABA-GE levels of 8-day-old seedlings grown on ½ MS media supplemented with different concentrations of ABA for 5 days (**E,F**; *n* = 40) and 11 days (**G,H**; *n* = 13). All data are given as mean ± standard deviation (SD) of six biological replicates. Double asterisks represent significant differences as determined by Student’s *t*-test at *P* < 0.01. **(I–L)** Transcript levels of chloroplast development-related genes grown on B5 media supplemented with DMSO or different concentrations of ABA for 1 day **(I)**, 3 days **(J)**, 5 days **(K)**, and 11 days **(L)**. Total RNAs were prepared from plants and used for qRT-PCR analysis. The expression levels with DMSO were set at 1.0. All data are given as mean ± SD (*n* = 3). Single and double asterisks represent significant differences as determined by Student’s *t*-test at *P* < 0.05 and *P* < 0.01, respectively.

Previous studies used widely varying ABA concentrations, from 1 to 100 μM, and the duration of ABA treatment varied from a few minutes to several days (Fujita et al., 2009; Park et al., 2009; Zhao et al., 2016). We subjected Arabidopsis plants to three different ABA concentrations (10, 50, and 100 μM), and monitored the effects over time. Treatment with 10 and 50 μM ABA induced leaf yellowing by 3 DAT, whereas 100 μM ABA induced much less pronounced leaf yellowing at 11 DAT (**Figures 2A–D**), indicating that ABA-induced leaf yellowing is not linearly dependent on ABA concentration.

Chlorophyll degradation is one of main causes of leaf yellowing (Lim et al., 2007). Therefore, we quantified the effect of ABA on leaf chlorophyll content. For this experiment, we tested the following ABA concentrations: 1 nM, 10 nM, 100 nM, 1 μM, 10 μM, 50 μM, and 100 μM. Plants were grown on ½ MS plates, transferred to ABA-supplemented plates, and then incubated for an additional 11 days. Plants treated with 1 or 10 nM ABA did not display any leaf yellowing (Supplementary Figure 2A). Consistent with the leaf color, chlorophyll contents were slightly higher in plants treated with 1, 10, or 100 nM ABA than in control plants. By contrast, chlorophyll contents were substantially lower in plants treated with 10, 50, and 100 μM ABA than in control plants (Supplementary Figure 2B). The chlorophyll contents were most severely reduced in plants treated with 10 μM ABA, which also induced the earliest onset of leaf yellowing.

Next, we assessed whether leaf yellowing was dependent on plant age. Plants grown for 7, 11, or 15 days were transferred to plates supplemented with 5 μM ABA, and examined for leaf yellowing at 10 DAT. Here we used 5 μM ABA instead of 10 μM to get an idea of the range of ABA concentration that effectively induces leaf yellowing. 5 μM ABA was as effective as 10 μM in inducing the yellow-leaf phenotype (Supplementary Figure 1). Moreover, these results indicate that ABA-induced leaf yellowing is not dependent on plant age up to 15 days.

### ABA Treatment Affects ABA Levels in Leaves

Previous work suggested that exogenous ABA should be imported from the medium into root cells, and then transported to the leaves (Sauter et al., 2001). However, it is not known how much ABA is taken up by the roots, or which form of ABA is transported from roots to leaves. For long-distance ABA transportation, UDP ABA-glucosyltransferases may generate ABA-GE, which is an inactive and membrane-impermeable form that can be transported from the root to leaves through the xylem (Jiang and Hartung, 2008). Therefore, we subjected plants to 10, 50, and 100 μM ABA and then measured cellular ABA levels at 5 and 11 DAT. The cellular ABA levels increased to higher levels proportionally to the plate ABA concentrations, but were only minor fractions of those in plates; 10, 50, and 100 μM ABA in plates resulted in cellular ABA concentrations of 0.058, 0.409, and 1.17 μg/g FW, respectively (**Figure 2E**). Given the same ABA concentrations in plates, the ABA levels in leaf tissues were slightly lower at 11 DAT than at 5 DAT (**Figure 2G**), indicating that cellular ABA levels did not increase with time. We also measured ABA-GE levels at 5 and 11 DAT. The cellular ABA-GE levels increased to proportionally higher levels depending on the plate ABA concentrations; 10, 50, and 100 μM ABA in plates resulted in cellular ABA-GE concentrations of 2.22, 13.90, and 33.98 μg/g FW, respectively (**Figure 2F**). Given the same ABA concentrations in plates, the cellular ABA-GE levels were higher at 11 DAT than at 5 DAT (**Figure 2H**). These combined results suggest that ABA imported into plants is actively converted to ABA-GE over time, and accumulates to higher levels with continuous exposure to exogenous ABA.

### Long-term ABA Treatment Disrupts the Expression of Genes Involved in Chloroplast Development

Next, we examined the effects of ABA on gene expression profiles over time. To correlate the gene expression patterns with the yellow-leaf phenotype, we focused on the following four genes involved in chloroplast development: *HEMA1*, *GLK1*, *PORA*, and *GUN4*. *HEMA1* is a glutamyl-tRNA reductase involved in 5-aminolaevulinic acid (ALA) synthesis, which is the first rate-limiting step in chlorophyll biosynthesis (McCormac et al., 2001). *GLK1* is a crucial transcription factor regulating the expression of genes involved in chloroplast biogenesis (Fitter et al., 2002; Waters et al., 2009). *PORA* is a light-dependent NADPH:Protochlorophyllide oxidoreductase, which generates chlorophyllide from protochlorophyllide (Tanaka et al., 2011). *GUN4* is a porphyrin-binding protein that regulates magnesium chelatase activity and is involved in plastid retrograde signaling (Larkin et al., 2003; Tanaka et al., 2011).

We transferred 8-day-old seedlings to plates supplemented with 10 or 100 μM ABA, and then examined gene expression at 1, 3, 5, and 11 DAT by preparing total RNA and performing qRT-PCR analysis. The expression levels of all four genes were strongly induced at 1 DAT by both 10 and 100 μM ABA (**Figure 2I**). For all four genes, the expression levels were much higher in seedlings treated with 10 μM than with 100 μM ABA (**Figure 2I**). The gene expression patterns changed dramatically at 3 DAT. At this time point, 10 μM ABA only slightly induced *GLK1* and *GUN4* expression levels but slightly suppressed *PORA* and *HEMA1* expression levels (**Figure 2J**). At 5 and 11 DAT, 10 μM ABA strongly suppressed the expression of all four genes, although *PORA* expression was slightly higher than that of the other three genes at both time points (**Figures 2K,L**). Treatment with 100 μM ABA induced more complex gene expression patterns. At 3 DAT, 100 μM ABA only slightly induced *PORA* and *HEMA1* expression but strongly induced *GLK1* and *GUN4* expression (**Figure 2J**). At 5 DAT, the expression levels of *PORA*, *GLK1*, and *GUN4* were induced by approximately twofold, whereas *HEMA1* expression was significantly suppressed (**Figure 2K**). At 11 DAT, *PORA* expression was slightly induced, whereas that of the other three genes was strongly suppressed (**Figure 2L**). These combined results suggest that ABA exerts complex effects on the expression of genes involved in chloroplast development. First, the exogenous ABA concentration and duration of treatment differentially affect the expression of genes involved in chloroplast development. Second, the observed ABA-induced gene expression patterns may depend on their exact functions in chloroplast development. Taken together, these results suggest that moderate ABA concentrations (in the range of 5–10 μM) of long-term duration more effectively inhibit the transcription of genes involved in chloroplast development, thereby contributing to leaf yellowing.

### ABA Treatment Differentially Affects the Expression of Genes Involved in Chloroplast Development Depending on the Duration of ABA Treatment

**FIGURE 3:**
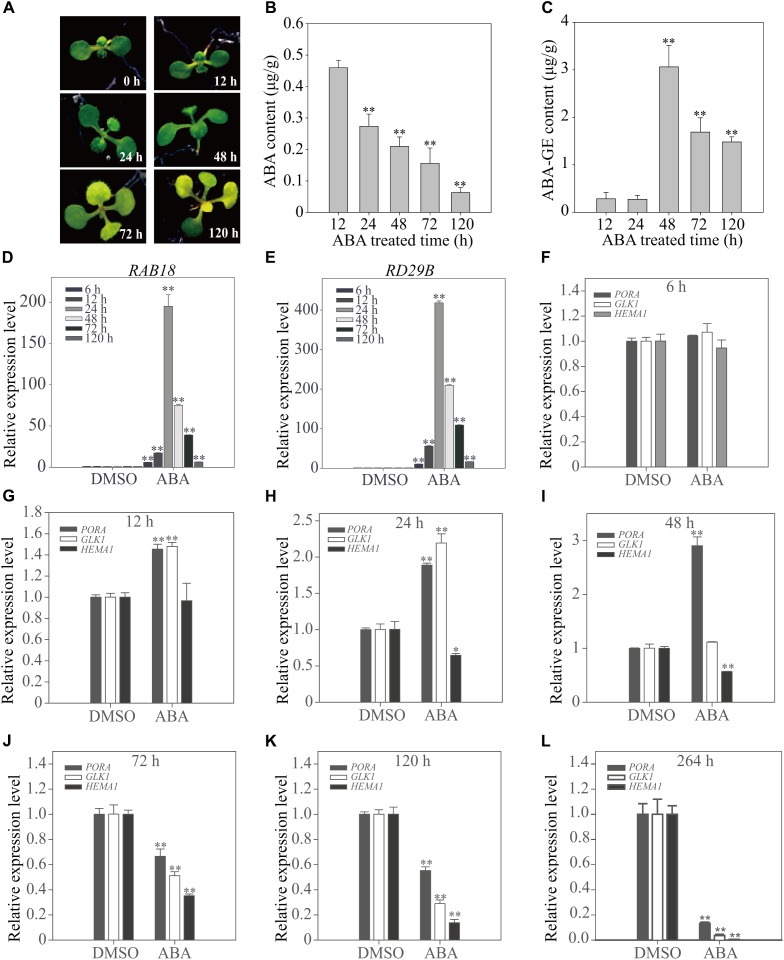
Chloroplast development-related genes show a transitional expression pattern upon exogenously applied ABA depending on the duration of treatment. **(A)** Phenotype of 8-day-old seedlings grown on ½ MS plates supplemented with DMSO or 5 μM ABA for the indicated periods of time. **(B,C)** Cellular ABA **(B)** and ABA-GE **(C)** levels of 8-day-old seedlings grown on ½ MS medium supplemented with 5 μM ABA for 12 h (*n* = 120), 24 h (*n* = 120), 48 h (*n* = 100), 72 h (*n* = 60) and 120 h (*n* = 50). Values represent mean ± SD of six biological replicates. Double asterisks represent significant differences as determined by Student’s *t*-test at *P* < 0.01. **(D,E)** Transcript levels of ABA-responsive genes of 12-day-old seedlings grown on ½ MS plates supplemented with DMSO or 5 μM ABA for the indicated periods of time. **(F–L)** Transcript levels of chloroplast development-related genes of 12-day-old seedlings grown on ½ MS plates supplemented with DMSO or 5 μM ABA for 6 h **(F)**, 12 h **(G)**, 24 h **(H)**, 48 h **(I)**, 72 h **(J)**, 120 h **(K)**, and 264 h **(L)**. All data in **(D–L)** are given as mean ± SD (*n* = 3). Single and double asterisks represent significant differences as determined by Student’s *t*-test at *P* < 0.05 and *P* < 0.01, respectively.

The expression pattern of genes involved in chloroplast development dramatically changes from activation to suppression depending on the duration of the ABA treatment (**Figure 2**). To obtain greater insight into the ABA-induced changes in gene expression patterns, we examined plants at early time points. Plants grown on ½ MS plates for 8 days were transferred onto ½ MS plates supplemented with 5 μM ABA, and then incubated for various periods of time (**Figure 3A**). First, we measured cellular ABA levels at 12, 24, 48, 72, and 120 h after transfer (HAT). Cellular ABA levels were gradually reduced from 0.46 μg/g FW at 12 HAT to 0.06 μg/g FW at 120 HAT (**Figure 3B**). We also measured cellular ABA-GE levels, which were approximately 0.3 μg/g FW at 12 and 24 HAT, drastically increased to 3.1 μg/g FW at 48 HAT, and then were maintained at 1.5–1.7 μg/g FW at later time points (**Figure 3C**). This indicates that the conversion of ABA to ABA-GE is highly activated at 48 HAT, but declines at the later time points.

The effect of ABA on plants was examined at the molecular level. Plants grown for 12 days on ½ MS plates were transferred to ½ MS plates supplemented with 5 μM ABA or DMSO as a control, and further incubated for additional periods of time. Total RNA was prepared and analyzed by qRT-PCR. First, we examined the expression patterns of the dehydration-related genes, *Responsive to ABA18* (*RAB18*) and *Responsive to Dessication29B* (*RD29B*), which are strongly induced in response to ABA treatment (Seki et al., 2002a,b). Both genes were strongly induced at early time points (6, 12, and 24 HAT) of ABA treatment, with maximal induction at 24 HAT, but the fold increase gradually declined with time, reaching two–threefold of that in control plants at 120 HAT (**Figures 3D,E**). Next, we examined the expression patterns of genes encoding the core components such as PP2Cs and cytosolic receptors of the ABA signaling pathway. Of the PP2C members, we selected three genes, *ABA Insensitive 1* (*ABI1*), *ABA Insensitive 2* (*ABI2*) and *Protein Phosphatase 2CA* (*PP2CA*) because they are known to be highly responsive to the ABA treatment (Santiago et al., 2009). Of 13 members of cytosolic receptors, PYR/PYL/RCARs, we selected three genes, *PYR-Like 4* (*PYL4*), *PYR1-Like 5* (*PYL5*) and *PYR1-Like 6* (*PYL6*), which are also responsive to ABA treatment (Santiago et al., 2009). Similar to the expression patterns of dehydration-related genes, *ABI1*, *ABI2*, and *PP2CA* were strongly induced at 24 HAT. However, the fold increase gradually declined with time. In contrast, the expression of *PYL4*, *PYL5*, and *PYL6* was suppressed from early time points of ABA treatment and further suppressed, although slightly, at later time points (Supplementary Figure 3). To gain insight into the leaf yellowing phenotype, we examined the expression pattern of the chloroplast development-related genes, *PORA*, *HEMA1*, and *GLK1*, at early time points after transfer to ABA plates. At 6 HAT, the expression of these genes was not significantly affected by ABA treatment (**Figure 3F**). At 12 HAT, the expression of *PORA* and *GLK1* was slightly induced, whereas the expression of *HEMA1* was not affected significantly (**Figure 3G**). At 24 HAT, the expression of *PORA* and *GLK1* was significantly induced, whereas the expression of *HEMA1* was significantly suppressed (**Figure 3H**). At 48 HAT, only *PORA* showed a threefold increase in transcript levels, whereas *GLK1* levels returned to those of the control and *HEMA1* levels declined (**Figure 3I**). At 72 and 120 HAT, the expression of all three genes was strongly suppressed (**Figures 3J–L**). These combined results indicate that short-term ABA treatment induces chloroplast-related gene expression, whereas long-term ABA treatment suppresses these genes. The expression patterns of these genes, in particular *PORA* and *GLK1*, were similar to those of osmotic stress-related genes, although the exact time point of the phase change from induction to suppression differed. Although the fold increases of these genes in **Figure 3** differed from those in **Figure 2**, which may be caused by differences in sucrose concentration, the trend of the expression pattern changes was similar to each other. Previous studies showed that sucrose affects ABA-mediated signaling (Finkelstein and Gibson, 2002).

**FIGURE 4:**
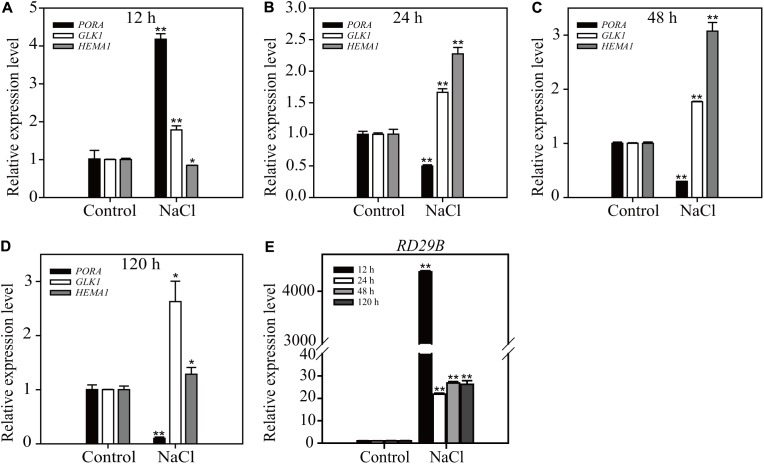
Salt stress induces a transitional expression pattern of chloroplast development-related and osmotic stress-responsive genes depending on the duration of treatment. Transcripts levels of chloroplast-related genes **(A–D)** and osmotic stress-related gene **(E)**. Eight-day-old seedlings were treated with 160 mM NaCl for the indicated periods of time. Total RNA was prepared from plants and expression levels were examined by qRT-PCR using gene-specific primer sets. *ACT2* was used as an internal control for qRT-PCR. All data are given as mean ± SD (*n* = 3). Single and double asterisks repr esent significant differences as determined by Student’s *t*-test at *P* < 0.05 and *P* < 0.01, respectively.

To access the physiological relevance of results obtained from long-term ABA treatment, we compared the effect of long-term NaCl treatment on the expression of chloroplast- and osmotic stress-related genes. ABA plays a crucial role in NaCl stress responses (Tuteja, 2007). Plants were treated with 160 mM NaCl for 12, 24, 48, or 120 h, and the expression levels of three chloroplast-related genes *PORA*, *HEMA1*, and *GLK1*, and one osmotic stress-related gene *RD29B* were examined by qRT-PCR. *PORA* and *RD29B* showed transitional responses; an initial strong induction at 12 h HAT followed by reduction in the induction levels at 24, 48, and 120 h HAT (**Figure 4**). Similarly the expression level of *HEMA1* displayed a transitional reponse from 12 to 120 h HAT. However, the expression level of *GLK1* was maintained at higher levels from 12 to 120 h HAT (**Figure 4**), which was different from the expression pattern of *GLK1* under the condition of long-term ABA treatment. One possible explanation is that high salinity has more profound effect than ABA treatment. Indeed, salt stress responses also include ABA-independent responses (Tuteja, 2007). These results support the idea that the effect of long-term ABA treatment may represnt a subset of physiological responses under the high osmotic stress.

### ABA Treatment Differentially Affects ABA Biosynthesis and Catabolism Depending on the Duration of ABA Treatment

**FIGURE 5:**
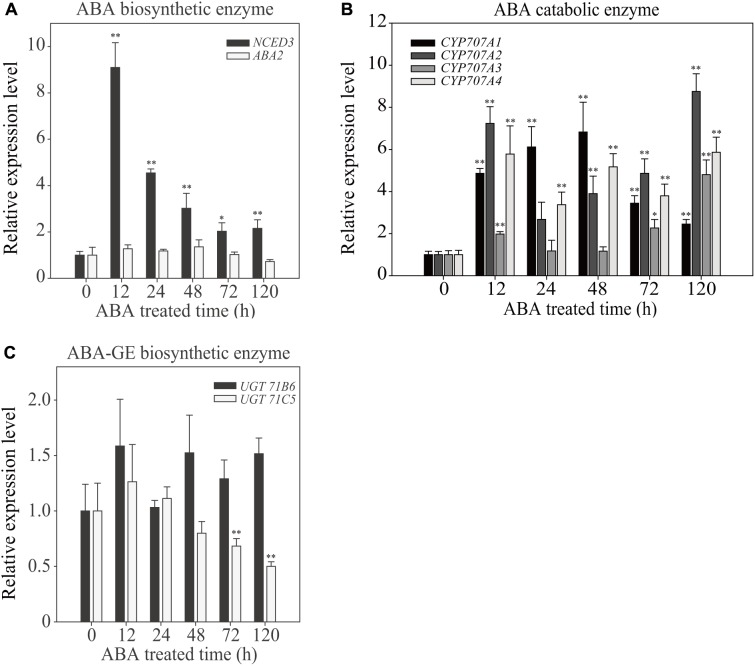
Effect of exogenously applied ABA on the temporal expression pattern of ABA biosynthetic and catabolic genes. Plants grown on ½ MS plates for 7 days were transferred onto ½ MS plates supplemented with DMSO or 5 μM ABA and grown further for the indicated periods of time. Total RNAs were prepared from plants and used for qRT-PCR analysis. Transcript levels of genes involved in ABA biosynthetic enzymes **(A)**, catabolic enzymes **(B)**, and UDP ABA-glucosyltransferases **(C)** were examined. *ACT2* was used as an internal control for qRT-PCR. All data are given as mean ± SD (*n* = 3). Single and double asterisks represent significant differences as determined by Student’s *t*-test at *P* < 0.05 and *P* < 0.01, respectively.

To gain insight into the effect of exogenous ABA on cellular ABA homeostasis, we examined the expression patterns of ABA biosynthetic and catabolic genes at different time points after ABA treatment for a period of 120 h. Exogenous ABA application affects the expression of genes involved in ABA production and degradation (Finkelstein, 2013). First, we examined the expression of two genes involved in *de novo* ABA biosynthesis, *9-CIS-EPOXYCAROTENOIDDIOXYGENASE 3* (*NCED3*) and *ABA DEFICIENT 2* (*ABA2*), which convert violaxanthin to neoxanthin and xanthoxin to abscisic acid aldehyde, respectively. In the ABA biosynthetic pathway, NCED is the rate-limiting enzyme (Nambara and Marion-Poll, 2005). Exogenously applied ABA increased the *NCED3* transcript level maximally to 9-fold at 12 HAT, but these levels gradually declined to a 2.5-fold increase at 120 HAT, indicating that the ABA biosynthetic pathway is highly activated at early time points of exogenous ABA treatment, but rapidly declines with time. The expression of *ABA2* was not affected by exogenous ABA (**Figure 5A**).

Next, we examined genes encoding ABA catabolic enzymes. ABA levels can be reduced by two different pathways, ABA hydroxylation by cytochrome P450-type hydroxylases, or conversion to the inactive ABA-GE by UDP ABA-glucosyltransferases (Kushiro et al., 2004; Priest et al., 2006; Finkelstein, 2013; Dong et al., 2014; Liu et al., 2015). First, we examined the expression of *CYP707A1*, *CYP707A2*, *CYP707A3*, and *CYP707A4*, which encode cytochrome P450-type hydroxylases. All four showed strong induction upon ABA treatment, with different temporal patterns. These genes were induced to 2.5- to 9-fold at 120 HAT depending on isoforms (**Figure 5B**), indicating that ABA hydroxylation is strongly activated by exogenous ABA. Next, we examined the induction pattern of two UDP ABA-glucosyltransferase genes, *UGT71B6* and *UGT71C5*. Overexpression of *UGT71B6* leads to high levels of ABA-GE (Priest et al., 2006), and a recent study showed that *UGT71C5* has an important role in ABA inactivation of ABA (Liu et al., 2015). Exogenously applied ABA had only mild effects on the induction of these genes (**Figure 5C**). *UGT71B6* showed approximately 1.5-fold induction at 120 HAT. By contrast, *UGT71C5* expression was suppressed with time. These results are not consistent with the results showing that the amount of ABA-GE was greatly increased upon exogenous ABA treatment. One possible explanation is that the glucose conjugation pathway of ABA does not respond significantly to exogenous ABA application at the transcription level but is modulated at the post-transcriptional level. We also cannot exclude the possibility that other isoforms of UDP ABA-glucosyltransferase genes may be activated at the transcription level.

### The Duration of ABA Treatment Is a Key Factor in Determining the Expression Pattern of Chlorophyll Degradation-Related Genes

**FIGURE 6:**
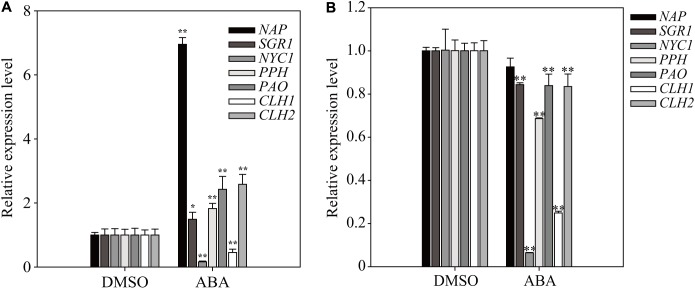
Exogenously applied ABA differentially affects the expression of chlorophyll degradation-related genes depending on the length of ABA treatment. Transcript levels of chlorophyll degradation-related genes were determined by qRT-PCR. Plants grown on ½ MS plates for 8 days were transferred onto ½ MS plates supplemented with DMSO or 5 μM ABA and grown further for 24 h **(A)** or 11 days **(B)**. All data are given as mean ± SD (*n* = 3). Single and double asterisks represent significant differences as determined by Student’s *t*-test at *P* < 0.05 and *P* < 0.01, respectively.

Chlorophyll degradation is a key step in the leaf-yellowing process, and several of the genes involved are under transcriptional control (Hortensteiner, 2009; Yang et al., 2014). To further elucidate the effect of short- and long-term ABA treatment on plant physiology, we examined the expression of some key genes involved in chlorophyll degradation, such as *STAY GREEN 1* (*SGR1*), *NONYELLOW COLORING1* (*NYC1*), *PHEOPHYTINASE* (*PPH*), *PHEOPHORBIDE a OXYGENASE* (*PAO*), *CHLOROPHYLLASE 1* (*CLH1*), and *CLH2* (Sakuraba et al., 2012). The *NAC-LIKE ACTIVATED BY AP3/PI* (*NAP*) transcription factor regulates chlorophyll degradation by promoting the transcription of *SGR1*, *NYC1*, *PPH*, and *PAO* (Yang et al., 2014). We prepared total RNA from plants grown on ABA-containing medium at 1 and 11 DAT. At 1 DAT, four genes (*SGR1*, *PPH*, *PAO*, and *CLH2*) were expressed at higher levels, and at 11 DAT, their expression was largely suppressed to varying degrees (**Figure 6**). By contrast, the dramatically induced expression of *NAP* at 1 DAT completely disappeared at 11 DAT. *NYC1* and *CHL1* were strongly suppressed at both 1 and 11 DAT. These results indicate that many chlorophyll degradation-related genes also exhibit a temporal transition in the expression pattern depending on the duration of ABA treatment.

### Long-term ABA Treatment Inhibits Chloroplast Division

**FIGURE 7:**
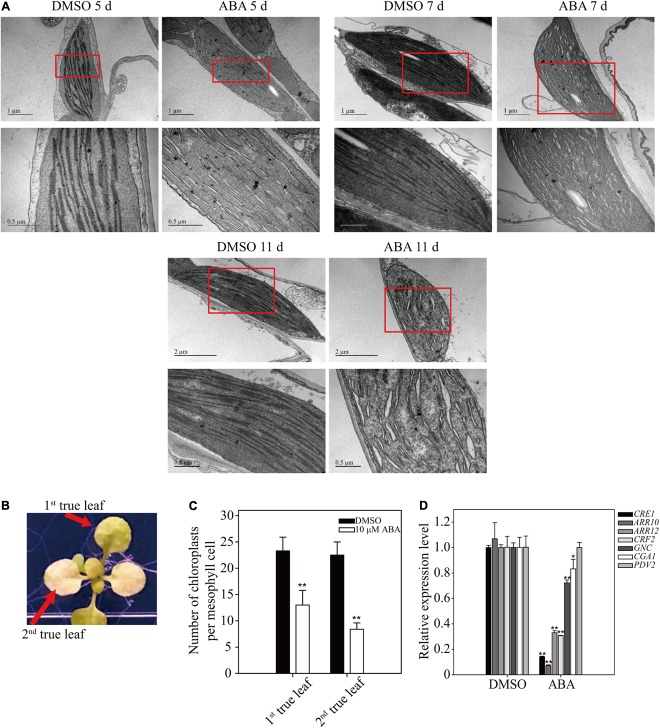
Effect of long-term ABA treatment on thylakoid development and chloroplast division. **(A)** Effect of exogenously applied ABA on chloroplast ultrastructure. Plants grown for 7 days on ½ MS plates were transferred onto ½ MS plates supplemented with DMSO or 5 μM ABA and further grown for the indicated periods of time. Ultrathin sections were prepared and used for transmission EM analysis. Lower images are enlarged images of the boxed areas. **(B,C)** Phenotype **(B)** and number of chloroplasts per mesophyll cell **(C)** of 10-day-old seedlings grown on ½ MS plates supplemented with DMSO or 10 μM ABA for an additional 15 days. All data in **(C)** are given as mean ± SD (*n* = 30). Asterisks and double asterisks in **(C)** represent significant differences as determined by Student’s *t*-test at *P* < 0.05 and *P* < 0.01, respectively. **(D)** Transcript levels of cytokinin-related genes involved in chloroplast division. Seven-day-old seedlings were transferred onto ½ MS plates supplemented with DMSO or 5 μM ABA and further grown for an additional 9 days. Transcript levels of *ACT2* were used as internal control for qRT-PCR. All data in **(D)** are given as mean ± SD (*n* = 3). Asterisks and double asterisks in **(C,D)** represent significant differences as determined by Student’s *t*-test at *P* < 0.05 and *P* < 0.01, respectively.

To gain insight into the yellow-leaf phenotype resulting from long-term ABA treatment, we examined chloroplast ultrastructure using electron microscopy (EM). Plants (8-day-old) were transferred to plates supplemented with 5 μM ABA or DMSO control, and grown for an additional 5, 7, or 11 days. Ultrathin leaf sections were prepared and analyzed by EM. In the control plants, thylakoids in chloroplasts were not fully developed into highly staked grana structures at 5 DAT, but were fully developed at 7 and 11 DAT. In ABA-treated plants, thylakoid membranes did not show the staked grana structure, but instead were dilated with a large luminal space, particularly at 11 DAT (**Figure 7A**), indicating that prolonged ABA treatment inhibits thylakoid development, which in turn results in the yellow-leaf phenotype.

The fact that ABA inhibits thylakoid membrane biogenesis prompted us to examine the number of chloroplasts after prolonged ABA treatment. Plants grown for 10 days were transferred onto ½ MS plates supplemented with 10 μM ABA, and grown for additional 15 days (**Figure 7B**). The number of chloroplasts was determined in the first and second true leaves. The chloroplast numbers were significantly reduced in both leaves upon long-term ABA treatment (**Figure 7C**), indicating that ABA inhibits the division of chloroplasts in leaf tissues. Cytokinin is a phytohormone that plays a key role in chloroplast division (Cortleven and Schmülling, 2015). Core components involved in the cytokinin signaling pathway include the cytokinin receptor CRE1/AHK4 and type-B responsive regulators (ARR10 and ARR12). The downstream transcription factors GNC and CGA1 regulate several aspects of chloroplast development and plastid division (Chiang et al., 2012). The transcription factor CRF2 increases the level of PDV2 protein, which is required for plastid division (Okazaki et al., 2009). To obtain supporting evidence for the inhibitory effect of ABA on chloroplast division, we examined the expression of these seven genes by qRT-PCR. Upon prolonged ABA treatment, these seven genes were divided into three groups depending on their expression patterns: *CRE1*, *ARR10*, *ARR12*, and *CRF2* were strongly suppressed; *GCN* and *CGA1* were marginally suppressed; and *PDV2* expression was not affected (**Figure 7D**). These results corroborate the finding that ABA treatment inhibits chloroplast division. One possibility is that ABA inhibits cytokinin signaling, which in turn leads to inhibition of chloroplast division. In fact, previous studies showed that ABA and cytokinin act antagonistically to each other in cellular processes (Lu et al., 2014).

**FIGURE 8:**
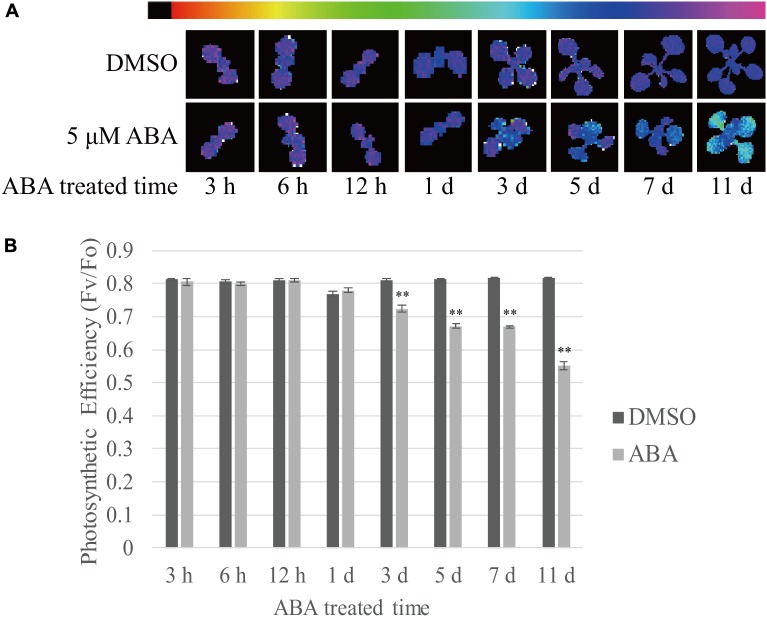
Prolonged exposure to ABA reduces the photosynthetic efficiency in plants. Plants grown on ½ MS plates for 8 days were transferred onto ½ MS plates supplemented with DMSO or 5 μM ABA and further grown for the indicated periods of time. **(A)** Chlorophyll fluorescence images were taken after dark adaptation for 30 min. The colored bar represents the values of *F*_v_/*F*_0_ ratio. The stronger purple indicates higher values of *F*_v_/*F*_0_ ratio. **(B)** To quantify photosynthetic efficiency, *F*_v_/*F*_0_ ratios were measured from the first true leaves. 12 first and second true leaves of 6 seedlings were analyzed and all data in **(B)** are given as mean ± SE. Double asterisks represent significant differences as determined by Student’s *t*-test at *P* < 0.01.

Finally, we measured the photosynthetic efficiency at various time points after ABA treatment. Photosystem II quantum yield (*F*_v_/*F*_0_ ratio) can be used as a simple indicator of photosynthetic efficiency, and this can be measured from chlorophyll fluorescence (Maxwell and Johnson, 2000). Stronger purple fluorescence indicates higher *F*_v_/*F*_0_ ratio. We observed that the *F*_v_/*F*_0_ ratio declined with time (**Figure 8A**). To quantify the photosynthetic efficiency, the *F*_v_/*F*_0_ ratio was measured using the first true leaves. The photosynthetic efficiency of control plants slightly increased with time during plant growth. By contrast, ABA treatment significantly reduced the photosynthetic efficiency to 68% of control levels at 11 DAT (**Figure 8B**). The reduction in photosynthetic efficiency was not as dramatic as the reduction in chlorophyll content (**Figure 1B**). These results confirm that ABA treatment affects photosynthetic efficiency by suppressing chloroplast biogenesis.

## Discussion

In this study, we investigated the temporal molecular and physiological responses to long-term ABA treatment. Exogenous ABA alone is known to induce many aspects of molecular and physiological responses that are induced by abiotic stresses (Tuteja, 2007). Thus, we reasoned that long-term ABA treatment may provide an insight into plant responses to the long-term abiotic stress.

We systematically investigated the temporal aspects of plant responses to exogenous ABA at both molecular and physiological levels. Upon treatment with exogenous ABA, the levels of cellular ABA increased rapidly. However, the ABA level peaked at 24 HAT followed by a gradual decline with time but was still higher than that in plants under the normal growth condition. It is not clear why the cellular ABA level decreased at later time points. One possibility is that the catabolic process is activated at the later time points. Indeed, four *CYP707As* involved in the hydroxylation of ABA were activated at the later time points. Another important ABA catabolic pathway is conversion of ABA to ABA-GE. It is possible that during long-term ABA treatment, ABA imported from the medium is actively converted to ABA-GE. Indeed, ABA-GE levels were higher upon ABA treatment. However, the contents of ABA-GE displayed a temporal transition in response to ABA treatment (**Figure 3C**). The contents of ABA-GE were maintained at low levels until 24 HAT but abruptly increased to higher levels at 48 HAT followed by a gradual decrease with time. However, the transcriptional regulation of the two genes we examined may not explain the delay in ABA-GE accumulation or abrupt accumulation of ABA-GE; the expression of *UGT71B6* was maintained at a slightly higher levels throughout the time course and the expression of*UGT71C5* was suppressed with time (**Figure 5C**). It is possible that the activities of ABA-GE biosynthetic enzymes may be modulated by post-transcriptionally. Another possibility is that other isoforms of ABA-GE producing genes may be regulated at the transcription level.

The effect of long-term ABA treatment was examined at the molecular level. The expression patterns of genes involved in dehydration stress responses, ABA biosynthesis, chloroplast development and chlorophyll degradation displayed a temporal transition in response to exogenous ABA; the expression of these genes was strongly induced at early time points such as 24 or 48 HAT, but induced at lower levels or suppressed at later time points. Thus, the mode of plant responses at the molecular level to exogenous ABA changes 24 or 48 HAT. The strong induction in the expression of many genes involved in dehydration stress or ABA metabolism at earlier time points is consistent with earlier reports (Seki et al., 2002a; Finkelstein, 2013). Indeed, exogenously applied ABA led to the increase in cellular ABA levels with a peak at 24 HAT. However, it is not clearly understood how the decline in induction fold or the suppression in the expression of these genes occurred at later time points even with the cellular ABA levels higher than that in plants under the normal growth condition. It is possible that the mode of ABA signaling is changed from a positive fashion to a negative fashion depending on the duration of higher cellular ABA levels. The negative mode of ABA signaling may function in turning off many cellular processes, thereby leading to the dormancy in plants. Another possibility is that the temporal difference in the levels of cellular ABA may be more important than the actual cellular ABA levels for initiating ABA-mediated signaling; upon ABA application, the ABA level peaked at 24 HAT and then declined with time, which renders inactivation of ABA signaling after 24 HAT, thereby resulting in the decline in induction levels of the gene expression. A previous study also showed that plant productivity displayed a transitional response to dehydration stress (Su et al., 2013). This transitional response consists of an acute phase at early time points and a prolonged phase at later time points (Su et al., 2013). The authors suggested that the earlier acute phase is needed for the drought-treated plants to reprogram reproductive development to enter the prolonged phase.

The most prominent phenotype caused by the long-term ABA treatment was the defect in chloroplast development, resulting in leaf yellowing. Among the concentrations of ABA we used, 10 μM ABA was most effective in inducing yellow leaf phenotype. In fact, when plants were grown on plates supplemented with 10 μM ABA, the amount of cellular ABA was 0.058 μg/g fresh weight (**Figure 2E**). Thus, this is comparable to the amount of cellular ABA that is 5 μg/g dry weight under the water-stressed (-0.3 MPa) conditions (Creelman et al., 1990). Upon exogenous ABA treatment, the expression of genes involved in chloroplast biogenesis was induced at early time points (**Figures 2**, **3**). A previous study showed that ABA plays a positive role in chloroplast biogenesis during early embryogenesis (Kim et al., 2009). However, long-term ABA treatment caused leaf yellowing due to defective chlorophyll biogenesis. These results raise the possibility that the duration of ABA treatment is critical in determining the physiological role of ABA in chloroplast biogenesis. Long-term dehydration stress also results in chlorotic leaves (Landi et al., 2016). As ABA levels are increased under dehydration stress conditions, it is possible that ABA plays a role in dehydration stress-induced leaf chlorosis. Leaf senescence may underlie the leaf chlorosis phenotype. Indeed, ABA is known to induce leaf senescence by inducing the expression of many senescence-related or chlorophyll degradation-related genes (Gao et al., 2016). Indeed, the expression of chlorophyll degradation-related genes was induced at early time points (**Figure 6A**), consistent with earlier studies showing that ABA activates chlorophyll degradation-related or senescence-related genes (Yang et al., 2014). However, chlorophyll degradation or senescence-related genes were suppressed at late time points of ABA treatment (**Figure 6B**), thus raising the possibility that leaf yellowing induced by long-term ABA treatment is caused by some other mechanisms. A recent study showed that ABA also plays a role in inhibition of dark-induced leaf senescence via a pathway involving ABI5-ABR (Su et al., 2016). In fact, the present study also showed that long-term ABA treatment did not induce senescence-related or chlorophyll degradation-related genes at late time points when the leaf yellowing phenotype was more clearly visible. Thus, leaf yellowing at the later time points may not be directly related to ABA-mediated activation of chlorophyll degradation or leaf senescence. Rather, prolonged ABA treatment may inhibit chloroplast development. Consistent with this idea, the expression of genes involved in chlorophyll biogenesis was strongly suppressed at later time points of ABA treatment (**Figures 2**, **3**). The expression of genes involved in cytokinin signaling also was strongly suppressed by long-term ABA treatment (**Figure 7D**). Cytokinin plays a critical role in chloroplast biogenesis and delays senescence (Zwack and Rashotte, 2013; Cortleven and Schmülling, 2015). Thus, one possible explanation is that prolonged ABA treatment actively blocks chloroplast biogenesis by inhibiting cytokinin signaling. Indeed, cytokinin and ABA act to each other antagonistically in certain cellular processes (Lu et al., 2014). Consistent with this idea, chloroplast division was suppressed by exogenous ABA (**Figure 7C**). Under the abiotic stress conditions such as dehydration or osmotic stress conditions, high chlorophyll content may be not favorable because light energy captured by the chlorophyll cannot be used to fix CO_2_, and instead results in high levels of ROS (Tripathy and Oelmülle, 2012). Thus, lowering the amount of chlorophyll is favorable under dehydration stress. Of course, we cannot rule out other possibilities because plants develop yellow leaves under many different conditions. One possibility is that the severe reduction in the genes involved in chloroplast biogenesis, thereby resulting in the yellow leaf phenotype, could be due to cell death caused by ABA toxicity when plants were treated with ABA too long (**Figures 2I–L**, **3J–L**). However, we are not in favor of this possibility; plants still grew, generated new leaves, and showed primary root growth during long-term ABA treatment, even though the growth rate was much slower in the presence of ABA than DMSO control. This suggests that even if the long-term ABA treatment may exert a certain degree of ABA toxicity to plants it may not be the main reason for the yellow leaf phenotype. Another possibility is that high levels of ABA inhibits import of resources such as iron needed for biogenesis of chlorophyll and chloroplasts (Ramírez et al., 2013; Li et al., 2014). However, the exact mechanism by which long-term ABA treatment induces leaf yellowing should be further studied in the future. By contrast to true leaves, young cotyledons remained largely green during long-term ABA treatment, raising the possibility that the effect of ABA on chloroplast biogenesis in cotyledons is different from that in true leaves.

What underlies the transitional responses to exogenously applied ABA? Because ABA is a key mediator of abiotic stress responses (Xiong and Zhu, 2003; Tuteja, 2007), the transitional response to exogenous ABA we observed in this study may represent a temporal pattern of plant responses to abiotic stress. Plants cannot predict how long abiotic stress conditions will last. Thus, one possible scenario is that plants continuously reprogram the responses to the abiotic stress according to the duration of the stress. Supporting this idea is that plants show acclimation or adaptation to stress conditions (Kinoshita and Seki, 2014). The transitional responses to the long-term ABA treatment imply that, at the early time points of abiotic stress such as dehydration or osmotic stresses, plants activate different cellular processes, some of which may even require more water supply to cope with the abiotic stress conditions. Indeed, the dehydration-induced genes are also induced by the long-term ABA treatment (Ingram and Bartels, 1996; Bray, 2004). The present study also provides evidence that plants enhance the expression of various genes, including those involved in chloroplast biogenesis, within 24 h of ABA treatment. This would support photosynthesis, a water-requiring process, and thereby produce more carbon sources, one of most valuable cellular resources necessary to cope with dehydration stress. However, if the abiotic stress such as dehydration stress continues then plants would need to change the mode of responses toward a survival strategy, in which the cellular activity needs to be minimized so that the use of water can be minimized. This could enable plants to survive until abiotic stress is relieved.

In summary, we provide evidence that plants show a temporal transition in responses to exogenously applied ABA. We propose that plants reprogram their responses to dehydration stress depending on the duration of the stress as shown by a temporal transition in responses to exogenously applied ABA. At the early phase, signaling involved in the abiotic stress responses is activated to turn on various cellular processes by increasing the expression of many genes, such as those involved in chlorophyll biogenesis, to maintain cellular activities. At the late phase, gene expression patterns are reprogramed to minimize the cellular activities and promote survival under prolonged dehydration stress conditions.

## Accession Numbers

Sequence data from this article can be found in the Arabidopsis Genome Initiative or GenBank/EMBL databases under the following accession numbers: PORA (At5g54190), HEMA1 (At1g58290), GLK1 (At2g20570), GUN4 (At3g59400), RAB18 (At5g66400), RD29B (At5g52300), CYP707A1 (At4g19230), CYP707A2 (At2g29090), CYP707A3 (At5g45340), CYP707A4 (At3g19270), NCED3 (At3g14440), ABA2 (At1g52340), UGT71B6 (At3g21780), UGT71C5 (At1g07240), NAP (At1g69490), SGR1 (At4g22920), NYC1 (At4g13250), PPH (At5g13800), PAO (At3g44880), CLH1 (At1g19670), CLH2 (At5g43860), ORE1 (At5g39610), SAG113 (At5g59220), CRE1 (At2g01830), ARR10 (At4g31920), ARR12 (At2g25180), CRF2 (At4g23750), GNC (At5g56860), CGA (At4g26150), PDV2 (At2g16070), PYL4 (AT2G38310), PYL5 (AT5G05440), PYL6 (AT2G40330), ABI1 (AT4G26080), ABI2 (AT5G57050), PP2CA (AT3G11410) and ACT2 (At3g18780).

## Author Contributions

IH devised and supervised the project and wrote the manuscript. MW and JL designed the experiments, analyzed the data, and performed the most experiments. MW and JL performed the most experiments. BC and YP conducted some physiological assays. H-JS and HK measured the ABA levels. MW, JL, and IH revised and approved the manuscript.

## Conflict of Interest Statement

The authors declare that the research was conducted in the absence of any commercial or financial relationships that could be construed as a potential conflict of interest.
