# Supplementary material for: Physiological and Molecular Processes Associated with Long Duration of ABA Treatment
Source: Front Plant Sci. 2018 Feb 21;9:176. doi: 10.3389/fpls.2018.00176 (PMC5826348; doi:10.3389/fpls.2018.00176)
Supplement: Supplementary file 1 [file Table_1.DOCX]

Supplementary Material

Physiological and molecular processes associated with long duration of ABA treatment

**Mei Wang^1,2#^, Juhun Lee^2#^, Bongsoo Choi^2^, Youngmin Park^2^, Hee-Jung Sim^4,5^, Hyeran Kim^6^, and Inhwan Hwang^2,3*^**

^1^ Key Laboratory of Plant Cell Engineering and Germplasm Innovation, Ministry of Education, School of Life Science, Shandong University, Jinan 250100, China

^2^ Division of Integrative Biosciences and Biotechnology and ^3^Department of Life Science, Pohang University of Science and Technology, Pohang 37673, Korea

^4^ Center for Genome Engineering, Institute for Basic Science, Yuseong-gu, Daejeon 305-811, Korea

^5^Environmental Toxicology Research Center, Gyeongnam Department of Environmental Toxicology and Chemistry, Korea Institute of Toxicology, 17 Jegok-gil, Munsan-eup. Jinju, Gyeongsangnam-do 52834, Republic of Korea

^6^ Department of Biology, College of Natural Sciences, Kangwon National University, Chuncheon 24341, Korea These authors contributed equally to this work.

^#^ These authors contributed equally to this work.

*** Correspondence:** Inhwan Hwang, ihhwang@postech.ac.kr

**
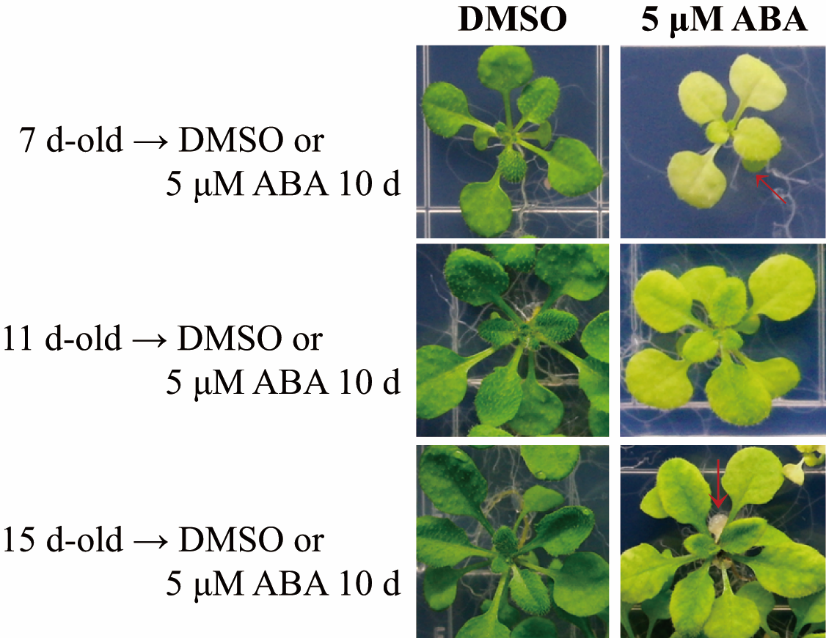
**

**Supplementary Figure 1.** **ABA-induced yellow-leaf phenotype is independent of plant age.** Plants grown on ½ MS plates for 7 days (**A**), 11 days (**B**), and 15 days (**C**) were transferred onto ½ MS plates supplemented with DMSO or 5 μM ABA, and further grown for an additional 10 days. Red arrows indicate cotyledons of the plant.


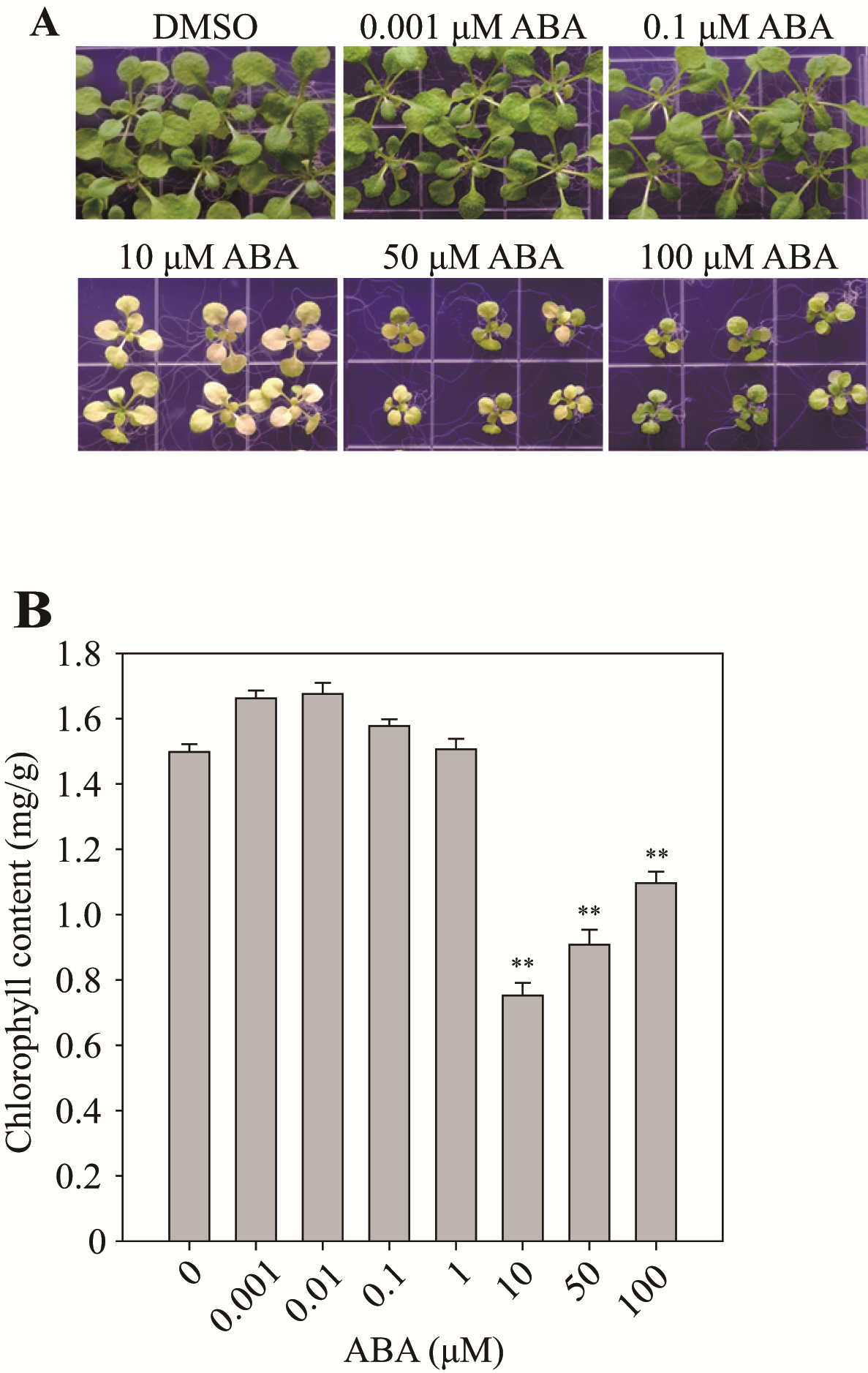


Supplementary Figure 2. Low and high concentrations of ABA differentially affect the chlorophyll contents. Phenotype (A) and chlorophyll content (B) of 8-day-old plants grown on ½ MS plates supplemented with DMSO or different concentrations of ABA for an additional 11 days.


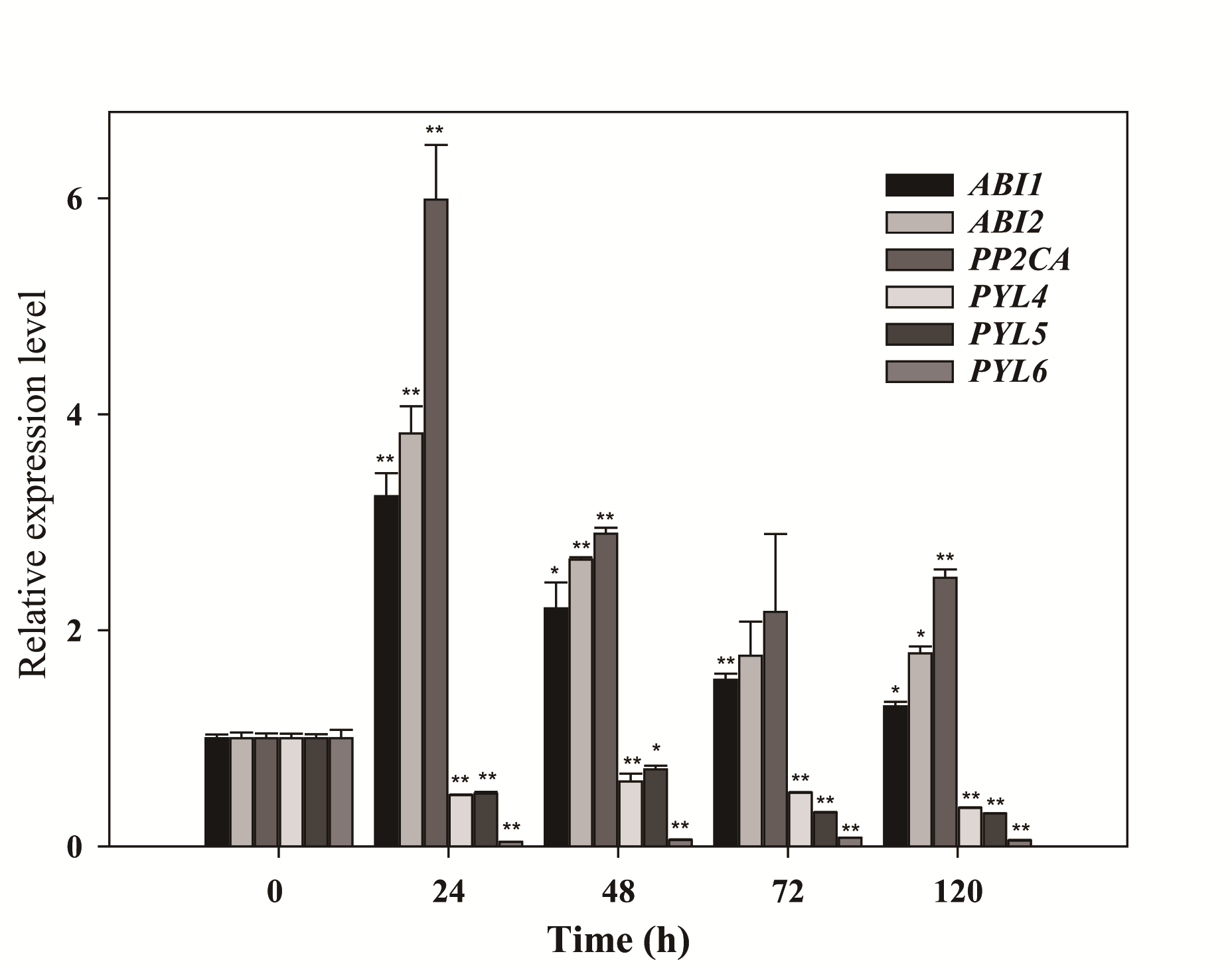


**Supplementary Figure 3.** Transcript levels of genes encoding the ABA signaling core components. 8 day-old plants (Col-0) were treated with 5 μM ABA for the indicated periods of time. Total RNA was prepared and used for qRT-PCR analysis using gene-specific primer sets.

**Supplementary Table 1. Nucleotide sequences of primers used in this study**

| qRT-PORA-F | CAACGACTGGTTTGTTCAGAGAGC |
| --- | --- |
| qRT-PORA-R | CAGCCACCACCTGAGCAAGTC |
| qRT-HEMA1-F | AGACGGTTCCAACGATCAAGA |
| qRT-HEMA1-R | CTCGAGCTCTGCCACTCTGA |
| qRT-GLK1-F | AGGCCGAAGCGGCTAATT |
| qRT-GLK1-R | GCACCGGTGTCTACTCCATA |
| qRT-GUN4-F | CGCCACAACTGCCGAAA |
| qRT-GUN4-R | TTGACGAGATGGTTCTCCAGA |
| qRT-RAB18-F | CGGGACTGAAGGCTTTGGA |
| qRT-RAB18-R | CACCACTTTCCTTGTGGAGTTG |
| qRT-RD29B-F | TTCTGACCACACCAAACCCAT |
| qRT-RD29B-R | CAGCCAGTGCCTCATGTCC |
| qRT-CYP707A1-F | AGCGCCGCTTTGGATCTT |
| qRT-CYP707A1-R | CGTAAGGCCAACCCATTGTT |
| qRT-CYP707A2-F | GCGGCTGGTCCAGTCTTCT |
| qRT-CYP707A2-R | TCGATGTGAGAGACGGTTGGT |
| qRT-CYP707A3-F | TCCTCCACGAAACTCCCTCTT |
| qRT-CYP707A3-R | TGTTTCGCCGACGTAAGGAT |
| qRT-CYP707A4-F | CACATTGCCCTTTCTTCCTTACA |
| qRT-CYP707A4-R | CCTGGTAGGTGGAGACAATCG |
| qRT-NCED3-F | TCTCCCGCCATTGTTGTTAAG |
| qRT-NCED3-R | CCGCTCTCTGGAACAAATTCA |
| qRT-ABA2-F | TTCTCTTCCTAGTCAAAGGCTTT |
| qRT-ABA2-R | GCAGACTTTGGCACCGTGCT |
| qRT-UGT 71B6-F | CGGTTGCTAAACTCGTAGATTCC |
| qRT-UGT 71B6-R | AACAACGAATCCCGCAAGAC |
| qRT-UGT 71C5-F | CGGGAGCATGGGAGTCTTC |
| qRT-UGT 71C5-R | CGAGCGCGTGAGCAATC |
| qRT-NAP-F | CGAAGCAGAGAGAAGAACTGAA |
| qRT-NAP-R | CAAATGAGCCAGCGAACAC |
| qRT-SGR1-F | GGGAAAATGTCGCTTCACG |
| qRT-SGR1-R | AGCCTTCAACACCACAGGTAG |
| qRT-NYC1-F | GCAGAGAACAGGACGAGGTT |
| qRT-NYC1-R | CGCAAACAACAGAAAGAGAGAA |
| qRT-PPH-F | AATCCCCAACGCTCCATACT |
| qRT-PPH-R | CTTCAAAACCACCAGACTCCA |
| qRT-PAO-F | CCCAGGCAGACCGTTTTGT |
| qRT-PAO-R | TGACTCTTACCATGCCGTCTGA |
| qRT-CLH1-F | CCCGTCGTTTTATTCTTCCA |
| qRT-CLH1-R | AGCATCGTCCACTTCCACTT |
| qRT-CLH2-F | ACAAAAGGGATTAGAGGGAAGAG |
| qRT-CLH2-R | GATACAACAAGTCCACCAACGA |
| qRT-CRE1-F | TCATGCTTTGGCTATTCTCG |
| qRT-CRE1-R | TCTCAAATGCTGTTCTTGCC |
| qRT-ARR10-F | TGCGTTCTGCGACTCAGCTA |
| qRT-ARR10-R | GAGATGCCGGATTTGTATCCA |
| qRT-ARR12-F | ACGGATGCTATGGCTCTGTTGAGT |
| qRT-ARR12-R | ACAAATCTCCCTGGCTCTGTTCCT |
| qRT-CRF2-F | CGTTGTAGTCAAGGAAGAGCCATC |
| qRT-CRF2-R | CGTACACATATCCGCCGTGAATAG |
| qRT-GNC-F | GCGTGATTAGGGTTTGTTCG |
| qRT-GNC-R | CTTTGCCGTATACCACATGC |
| qRT-CGA1-F | CCATATCTCCCAACCTCTCG |
| qRT-CGA1-R | TGGGCACCATTTGATCACT |
| qRT-PDV2-F | GCATCACGAATACGCAAAAGC |
| qRT-PDV2-R | GATCTTTCTCCCGGAGAAGGAA |
| qRT-ACT2-F | TATGAATTACCCGATGGGCAAG |
| qRT-ACT2-R | TGGAACAAGACTTCTGGGCAT |
